# A Decision Support System to Enhance Self-Management of Low Back Pain: Protocol for the selfBACK Project

**DOI:** 10.2196/resprot.9379

**Published:** 2018-07-20

**Authors:** Paul Jarle Mork, Kerstin Bach, SelfBACK Consortium

**Affiliations:** ^1^ Department of Public Health and Nursing Norwegian University of Science and Technology Trondheim Norway; ^2^ Department of Computer Science Norwegian University of Science and Technology Trondheim Norway

**Keywords:** mHealth, eHealth, case-based reasoning, digital health, machine learning, computer technology, smartphone, primary care, physical activity, exercise

## Abstract

**Background:**

Low back pain (LBP) is a leading cause of disability worldwide. Most patients with LBP encountered in primary care settings have nonspecific LBP, that is, pain with an unknown pathoanatomical cause. Self-management in the form of physical activity and strength and flexibility exercises along with patient education constitute the core components of the management of nonspecific LBP. However, the adherence to a self-management program is challenging for most patients, especially without feedback and reinforcement. Here we outline a protocol for the design and implementation of a decision support system (DSS), selfBACK, to be used by patients themselves to promote self-management of LBP.

**Objective:**

The main objective of the selfBACK project is to improve self-management of nonspecific LBP to prevent chronicity, recurrence and pain-related disability. This is achieved by utilizing computer technology to develop personalized self-management plans based on individual patient data.

**Methods:**

The decision support is conveyed to patients via a mobile phone app in the form of advice for self-management. Case-based reasoning (CBR), a technology that utilizes knowledge about previous cases along with data about the current patient case, is used to tailor the advice to the current patient, enabling a patient-centered intervention based on what has and has not been successful in previous patient cases. The data source for the CBR system comprises initial patient data collected by a Web-based questionnaire, weekly patient reports (eg, symptom progression), and a physical activity-detecting wristband. The effectiveness of the selfBACK DSS will be evaluated in a multinational, randomized controlled trial (RCT), targeting care-seeking patients with nonspecific LBP. A process evaluation will be carried out as an integral part of the RCT to document the implementation and patient experiences with selfBACK.

**Results:**

The selfBACK project was launched in January 2016 and will run until the end of 2020. The final version of the selfBACK DSS will be completed in 2018. The RCT will commence in February 2019 with pain-related disability at 3 months as the primary outcome. The trial results will be reported according to the CONSORT statement and the extended CONSORT-EHEALTH checklist. Exploitation of the results will be ongoing throughout the project period based on a business plan developed by the selfBACK consortium. Tailored digital support has been proposed as a promising approach to improve self-management of chronic disease. However, tailoring self-management advice according to the needs, motivation, symptoms, and progress of individual patients is a challenging task. Here we outline a protocol for the design and implementation of a stand-alone DSS based on the CBR technology with the potential to improve self-management of nonspecific LBP.

**Conclusions:**

The selfBACK project will provide learning regarding the implementation and effectiveness of an app-based DSS for patients with nonspecific LBP.

**Registered Report Identifier:**

RR1-10.2196/9379

## Introduction

### Background

The recent Global Burden of Disease Study showed that low back pain (LBP) is the most significant contributor to years lived with disability worldwide [1,2]. Accordingly, LBP is one of the most common reasons for activity limitation, sick leave, and work disability [3,4]. In addition to the suffering of affected individuals, LBP poses an enormous economic burden on society, presenting a huge challenge for health care systems.

selfBACK addresses nonspecific LBP, that is, pain with an unknown pathoanatomical cause, which comprises >85% of all patients with LBP observed in primary care settings [5,6]. In 2006, a European expert working group developed evidence-based guidelines for the management of nonspecific LBP [7,8]; these guidelines have subsequently been adopted and refined by several countries to outline the best practice and appropriate advice to manage LBP [9-13]. Although some variations exist, the main components recommended in the management of LBP include education and reassurance, staying active both in and outside of work, and regular strength and flexibility exercises to prevent relapse, pain-related disability, and chronicity.

Many patients with long-term conditions find it challenging to self-manage their illness, for example, through lifestyle modifications, with little or no additional support [14], and the adherence to self-management programs is commonly poor [15]. Thus, mobile technologies have been suggested as a promising approach to improve self-management of various health conditions [16,17]. In particular, the possibility of delivering tailored support to individual patients has a significant potential with some evidence that tailoring the self-management advice to patients with LBP is more effective compared with nontailoring [18]. Furthermore, increasing evidence suggests that “tailoring” of digital health products is an important factor likely to promote uptake and utilization [19]. However, further research is warranted to clarify how the tailoring of advice for self-management can be integrated and delivered with mobile technologies to promote self-management of LBP. Recent reviews have shown that nearly 300 pain-related mobile phone apps are available [20-23]; however, few of these apps have been developed with evidence-based content and not have they been rigorously tested for effectiveness on pain-related health outcomes [20-23]. Furthermore, health care professionals and patients have seldom been involved in the app development. Thus, a clear need exists for further research aimed at developing high-quality, effective, and smart self-management interventions for LBP [24,25].

In this paper, we outline a comprehensive protocol for the design and implementation of an evidence-based decision support system (DSS), selfBACK, which has the potential to improve self-management of nonspecific LBP. The core of selfBACK is to (1) provide effective evidence-based advice on physical activity and tailored exercise training according to personal goals, personal characteristics, symptom progress, and functional ability and (2) provide educational material to individuals on self-management of their LBP condition. The resulting selfBACK system constitutes a data-driven, predictive DSS that uses the case-based reasoning (CBR) methodology [26-28] to capture and reuse patient cases to suggest the most suitable self-management plan (ie, decision support) for an individual patient. Furthermore, structured intervention mapping will be conducted as an integrated part of the project to guide the design, development, and evaluation of the selfBACK app [29].

### Aim and Objectives

**Figure 1:**
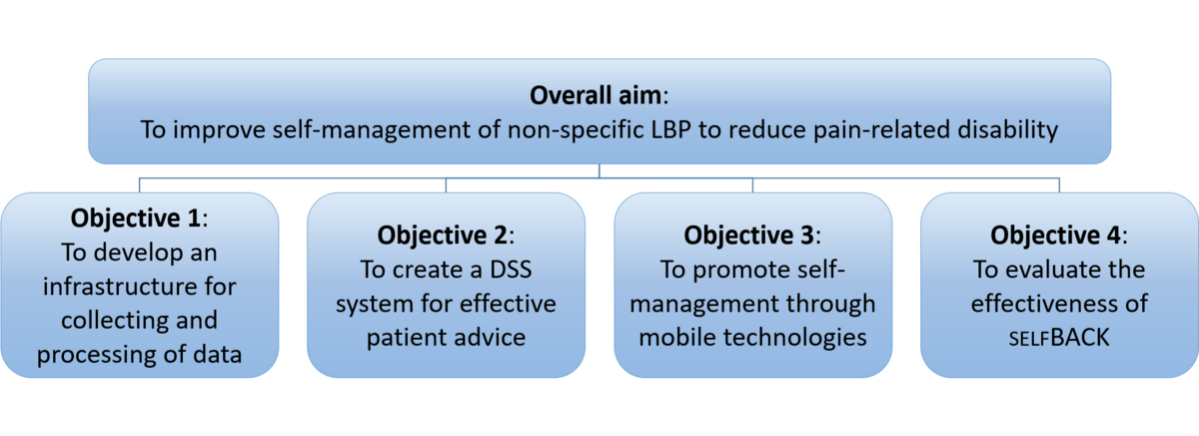
Overview showing how the overall aim is achieved by the selfBACK objectives. DSS: decision support system; LBP: low back pain.

The selfBACK project is a Research and Innovation Action funded under the Societal Challenges—Health, Demographic Change, and Well-Being call of the Horizon 2020 program. The project runs from the start of 2016 until the end of 2020. The overall aim of the selfBACK project is to improve self-management of nonspecific LBP to reduce pain-related disability. Figure 1 provides an overview of the project objectives. *Phase 1* of the project comprises the development and implementation of the selfBACK system (objectives 1-3) and will run until the end of 2018. *Phase 2* of the project comprises a randomized controlled trial (RCT) to evaluate the effectiveness of selfBACK (objective 4).

#### Objective 1—To Develop an Infrastructure for Collecting and Processing Data

The self-management plan will be tailored to each patient according to data collected by a baseline Web-based questionnaire, a weekly question and answer (Q/A) session in the selfBACK app, and a physical activity-detecting wristband worn by patients. Besides developing an infrastructure for collecting and processing these data, objective 1 also includes work that focuses on the definition of case representations and similarity measures, which are the core components of the CBR technology.

#### Objective 2—To Create a Decision Support System for Effective Patient Advice

Specifically, the selfBACK system is designed to assist patients in deciding upon and reinforcing the appropriate actions to manage their LBP. Based on the current best evidence, specific content for supporting physical activity, patient education, and strength and flexibility exercises are developed as part of the DSS. Besides CBR for handling situation-specific knowledge, elements of the model- and rule-based reasoning are used to capture and utilize generalized knowledge (eg, clinical guidelines) as well as customize the recommendations for self-management.

#### Objective 3—To Support Self-Management Through the selfBACK App

The decision support is conveyed to patients by a mobile phone app. The app provides patients with (1) instant feedback on the activity level and activity distribution (based on the data stream from the wristband) in accordance with the personal goals set by them and (2) tailored educational sessions and specific exercise training in line with patients’ goals, personal characteristics, symptom progress, and functional ability.

#### Objective 4—To Evaluate the Effectiveness of the selfBACK System

The effectiveness of the selfBACK app will be evaluated in a multinational RCT (parallel group trial) that will target care-seeking patients with nonspecific LBP. The comparator will be patients who receive the usual treatment. In addition, a process evaluation will be carried out as an integrated part of the RCT to document barriers and facilitators for the uptake and utilization of the selfBACK app and for gaining an understanding of patient experiences of using the app. Furthermore, a detailed protocol of the RCT will be reported in a separate publication.

## Methods

### Concept and Approach

The concept underlying the design of the selfBACK system is that an improved clinical outcome in patients with LBP will rely on behavioral change, which, in turn, might be conditioned by several factors, such as fear avoidance, pain self-efficacy, comorbidities, mood, and physical and mental capacity. The literature offers numerous health behavioral change theories that explain and predict the physical activity behavior, several of which focus on the motivation and volition, in other words, intending to be active and transforming the intention into action [30]. In people suffering from LBP, several factors will affect the relationship between intention and behavior. For example, fear-avoidance beliefs have been demonstrated to affect the activity levels of people with LBP [31,32]. Moreover, it has been suggested that pain self-efficacy is important in mediating the relationship between pain and functional disability [33], and it has been recognized as one of the main drivers toward positive outcomes [34].

In the following section, we describe the technical solution that will be implemented to address the project objectives and concept underlying the selfBACK DSS.

### Components of the selfBACK System

**Figure 2:**
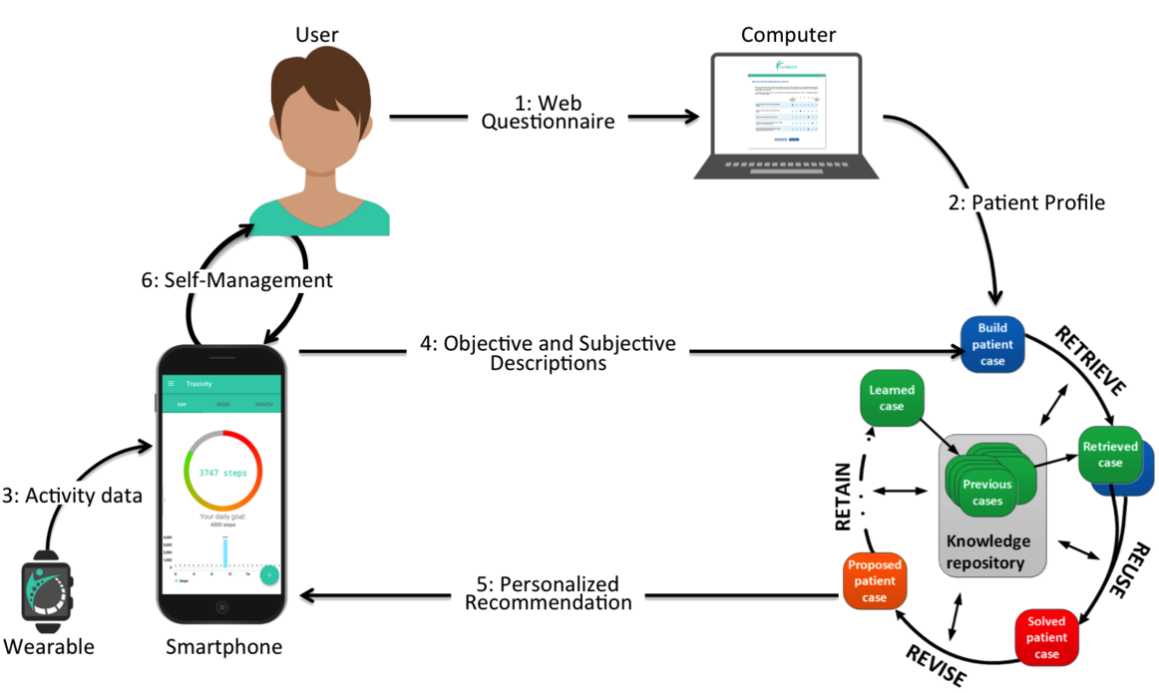
Illustration of the overall architecture and how the data processing of the person-directed modules link together in the selfBACK system.

The core of selfBACK is a DSS that helps patients to follow a plan for physical activity (ie, daily step count), education, strength, and flexibility exercises according to personal goals, personal characteristics, symptom progress, and functional ability. To accomplish this, selfBACK incorporates existing knowledge (eg, clinical guidelines and medical ontologies) and information provided by patients to recommend tailored advice for self-management. Figure 2 shows the overall architecture and basic modules of the selfBACK system.

**Figure 3:**
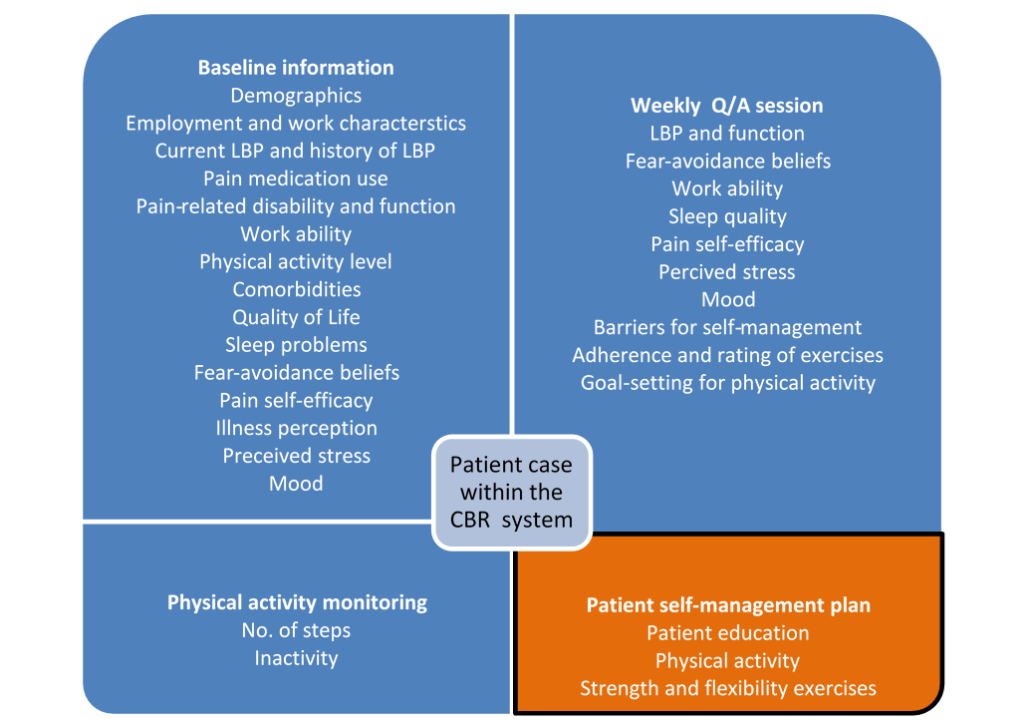
Components of a case description within the case-based reasoning (CBR) system containing the patient characteristics and the compiled advice. Only a relevant sub-set of the weekly questions will be asked in each session. The questionnaires used to collect baseline information are described in Table 1. LBP: low back pain; Q/A: question/answer.

The decision support is conveyed to patients via a mobile phone app in the form of advice for self-management. The app will be developed for Android and iOS using the React Native framework. The process for producing and tailoring the self-management plans and is illustrated by steps 1-6 in Figure 2. Before starting to use the selfBACK app, patients will be required to fill a Web-based questionnaire (1) that provides information about a range of personal characteristics that are used for tailoring the self-management plan (Figure 3, top left quadrant)—this information is fed to the selfBACK server; (2) to initiate the first CBR decision support cycle and produce the first self-management plan, which is pushed to the mobile phone (5) and accessed by patients (6). All further interactions happen via the mobile phone, which collects subjective tailoring data from patients on a weekly basis (4) as well as physical activity data from a wearable (3). The user interaction (4) is a Q/A module used to adjust the weekly self-management plan based on responses to questions on LBP, functional ability, fear avoidance, work ability, barriers for self-management, pain self-efficacy, sleep, perceived stress, mood, and adherence to the self-management plan. The only goal is to ask questions that are relevant for updating the current decision support for patients and avoiding unnecessary repetition of questions or questions not relevant for the follow-up of a particular patient.

For example, reporting of poor sleep will initiate an educational session targeting sleep behavior accompanied by appropriate follow-up questions. In contrast, reporting of good sleep will reduce the frequency of asking about sleep. Moreover, the regular monitoring of factors such as fear avoidance and pain self-efficacy by the weekly Q/A sessions allows us to make decisions about the amount and type of feedback and educational guidance required by individual users of the selfBACK app. For example, patients with low fear avoidance and high pain self-efficacy may only require simple educational follow-up information provision, whereas patients with high fear avoidance and low pain self-efficacy would require more support and guidance on behavioral change techniques, such as goal setting, pacing, and action planning. In addition, the activity data (3) provide information about whether patients follow the suggestions in the self-management plan (such as the number of steps per day). Regarding the baseline and Q/A data, activity log data are also sent to the selfBACK server giving input to the periodic run of the CBR cycle to generate an adjusted plan for self-management. Furthermore, this means that patients will receive a general advice on physical activity during the first week and that the goal for the number of steps per day will be adjusted in the consecutive weeks according to the incoming data from the wearable.

In addition to the follow-up and feedback on the daily step counts, the selfBACK system provides patients with recommendations on specific strength and flexibility exercises. The exercise plan conveyed to patients includes instructions and illustrations of the exercises along with recommendations for the number of sessions per week and repetitions or sets within a session. A typical exercise plan is designed to target strengthening of the back extensors, abdominals, gluteal muscles, and core muscles along with the flexibility of knee and hip and trunk muscles with short videos demonstrating the execution of the exercises. At present, insufficient evidence exists to make strong recommendations for or against any specific strength or flexibility exercises [10,35]. Therefore, we will implement a codecision approach where the system suggests an exercise plan that patients can adjust if desired, for example, patients can select exercises they enjoy or feel are beneficial and report on the progression. We hypothesize that this system will increase the adherence to the exercise plan and make it more likely that patients will sustain engagement with the selfBACK app over time. Besides the regular strength and flexibility exercises, the system will recommend pain-relief exercises in the case of a flare-up of symptoms. The latter is part of a “first-aid” kit that patients will have available, which will also include advice about other appropriate measures for acute LBP.

### The Decision Support System

The selfBACK system constitutes a data-driven, predictive DSS that uses the CBR methodology to capture and reuse patient cases to suggest the most suitable self-management plan for an individual patient [36]. The CBR cycle includes four processes that interact with a knowledge repository to suggest a personalized plan for self-management (Figure 2). The data collected by the baseline Web-based questionnaire, the Q/A session, and the wearable are formatted to match existing representations. Hence, we will build an individual patient case from this data, which subsequently would be matched with the existing case base (RETRIEVE). Then, the best matching case will be selected to be fitted to the current patient (REUSE). A core method in the retrieval step is similarity assessment, which compares how similar cases are to each other on demographic, pain, and mood-related information. After retrieving a similar past successful case, a plan for self-management will be adapted to the current case. This process is guided by a set of adaption rules and the goals set by patients. The result is a personalized and individually tailored self-management plan for patients. The plan is fetched from the server by patients’ mobile phones as an *active case* and monitored over the planned time period. In addition, the REVISE step in the CBR cycle addresses the evaluation and possible revision of the output from REUSE before learning from the new case takes place. In the selfBACK system, this step is not performed at once but rather postponed until the effect of the system’s currently suggested plan (ie, the output from REUSE) can be assessed. At that time, patient's case might be stored as a new case in the knowledge repository (RETAIN). Furthermore, the case can be temporarily stored in a preliminary case store, where all cases whose effects have not yet been evaluated are kept. In general, all cases stored in the knowledge repository (learned cases) are available for subsequent development of self-management plans and activity suggestions, thus restarting the CBR cycle.

Figure 3 describes the data sources and the structural contents of a case in the selfBACK’s CBR system. The accompanying Table 1 provides an overview of the information collected at the baseline that was fed into the selfBACK DSS. The case consists of a patient’s description and matching self-management plan. Data for the patient’s description are acquired with different frequencies in the selfBACK lifecycle, and the self-management plan is updated accordingly. A substantial part of the information obtained at the baseline is static (eg, demographics), whereas other information is expected to vary over time and hence, will be updated on a regular basis through the Q/A session (eg, pain-related disability and function). In addition, the selfBACK system uses data abstractions, rather than raw data, for comparing cases. Each case in the case base is structured as illustrated in Figure 3. Every query is represented in the same way as a case but without a self-management plan; this is referred to as an input case. For the similarity-based comparison between an input case and past cases, the DSS runs three parallel services, which are as follows: (1) one taking all physical activity features into account to suggest activity goals; (2) the second service taking all strength and flexibility exercise features into account to suggest a new exercise program; and (3) the third service takes all relevant education features into account to suggest new education sessions.

**Table 1:** Overview of the information collected at the baseline.

Baseline information			Response options or questionnaire reference
Demographics			Age, gender, height, weight, family, ethnicity, education, employment status
Physical work characteristics			Saltin–Grimby Physical Activity Scale [37]
**Current LBP^a^**			
	Average last week	Visual analog scale, 0-10 [38]	
	Worst last week	Visual analog scale, 0-10 [38]	
**History of LBP**			
	Length of current episode	<1 week; 1-4 weeks; 5-12 weeks; >12 weeks	
	Days with LBP past year	0 days; 1-7 days; 8-30 days; >30 but not every day; every day	
Use of pain medication last week (days)			None; 1-2 days; 3-5 days, daily
Pain-related disability			Roland-Morris Disability Questionnaire [39]
Function			Patient Specific Functional Scale [40]
**Activity limitation**			
	Reduced work activity	Yes; no	
	Reduced leisure time activity	Yes; no	
Current work ability			Work ability index, 0-10 (single item) [41]
Leisure time physical activity			Saltin–Grimby Physical Activity Scale [37]
Comorbidities, musculoskeletal			Pain mannequin
Comorbidities, others			Cardiovascular disease; heart failure; stroke or brain hemorrhage; asthma; chronic bronchitis or emphysema, COPD^b^; diabetes; gastrointestinal problems; kidney disease; cancer; epilepsy; osteoporosis; osteoarthritis; depression; anxiety; sleep apnea; rheumatoid arthritis; psoriatic arthritis or psoriasis; other
Quality of life			EQ-5D^c^ [42]
Sleep problems			Sleep Screening Questionnaire [43]
Fear-avoidance beliefs			Fear-Avoidance Beliefs Questionnaire [44]
Pain self-efficacy			Pain-Related Self-Efficacy Questionnaire [45]
Illness perception			Brief Illness Perception Questionnaire [46]
Perceived stress			Perceived Stress Scale [47]
Mood			Patient Health Questionnaire [48]

^a^LBP: low back pain.

^b^COPD: chronic obstructive pulmonary disease.

^c^EQ-5D: European Quality of Life-5 Dimensions.

### The selfBACK Architecture

The selfBACK architecture consists of clients that are connected to a server, which holds the DSS. The architecture covers the following two main scenarios. First, a patient is provided with the necessary credentials to access a web interface to be used for the initial sign-up and to fill out a baseline questionnaire, and second, a patient is equipped with a wearable and the selfBACK mobile phone app, which guides self-management according to the goals set by the patient. The mobile phone app synchronizes with the wearable and obtains the patient’s activity log and sends push notifications with content that encourages physical activity. Furthermore, the mobile phone app itself is the tool for the patient to obtain personalized information and educational explanations.

The decision support server performs all relevant tasks for maintaining the knowledge repository and provides the infrastructure for the advice generation services. Those services are parts of the server and communicate with clients through a secure access layer. The data generated will be used for updating each patient’s self-management plan.

The clients are either native mobile phone app or web browser users accessing the selfBACK system. The server can only be accessed through the secure access layer, which requests authentication and maintains user groups and secure workflows ensuring only relevant data are accessible to any client. In addition, the decision support server performs all relevant tasks from preprocessing incoming data and creating abstractions to running the decision support engine; this process is enhanced by machine learning tasks, which enable the creation of new cases and rules from incoming data. The knowledge obtained from this process is stored permanently in the knowledge repository.

The core of the DSS is the CBR system for finding the most similar patient cases. An additional rule-based system module captures generalized knowledge that is complementary to the situation-specific knowledge in cases in the form of clinical guidelines and generalizations over cases. Depending on its type, each incoming data stream is preprocessed in a particular way. In addition, data abstraction enhances the incoming data with domain knowledge, thereby allowing more comprehensive reasoning to support the decision making. Furthermore, implementing case matching as software services allows selfBACK to scale and perform the generation of self-management plans in parallel, whereas the core engine and knowledge repository stay consistent.

The knowledge repository holds cases and rules as well as the underlying ontology, and provides this knowledge for the DSS. The case base, containing cases, is the primary source of knowledge. Rules provide additional knowledge, particularly targeted for representing relevant clinical guidelines and knowledge. Furthermore, the ontology defines the concepts used by cases and rules with essential relations such as the taxonomical relations that enable the inheritance inference within the ontology.

### Data Processing in selfBACK

Preprocessing is performed on all data sources, which are included in the target knowledge models. Data are structured and grouped according to the domain and information types using the ontology. In addition, incoming data are cleaned, normalized, and transformed, and the significant features and instances are selected. The preprocessing strategy depends on the source data and its purpose in the knowledge model.

Data abstraction is applied to make data streams comparable and prepare data for the next process, the personalized decision support. selfBACK uses existing state-of-the-art methods for detecting trends in raw data. In addition, the individual activity streams are processed to reduce complexity or enhance them with the information required for better matching. Once data are delivered to the DSS, each patient is represented as a self-management agent who uses the incoming data to build up a query (a case with a problem description only) and match it against the case base. Consequently, it receives the best matching case that is subsequently personalized to give appropriate advice, enhanced with explanations. Then, an updated self-management plan is returned to the user. Explanations might be justifications for the advice, that is, “how was this advice derived?,” and an explanation of the effects of the advice given the current situations of patients. Furthermore, explanations are stored as predefined text elements in a separate part of the knowledge repository.

### Structured Intervention Mapping and User Involvement

In this study, structured intervention mapping is used to guide the development of the content for the selfBACK system [29]. Importantly, structured intervention mapping promotes a strong theoretical underpinning of the logic model of an intervention. In selfBACK, the logic model is underpinned by behavioral change theories [49] and the normalization process theory [50] to help us understand and evaluate the factors that promote or inhibit the uptake, utilization, and sustained use of the selfBACK app. Moreover, developing selfBACK through the involvement of users and key stakeholders (ie, patients and clinicians) maximizes sustainability and empowerment, increases commitment to the intervention, and increases credibility and likelihood of the uptake and utilization of the intervention [29]. In addition, direct input from patients, through primary research methods like observation, interviews, and focus groups, provides insights into users’ behavior, including what they want to do with the selfBACK app, how the selfBACK app is integrated into their living environment, when and how they will use it as well as the perceived barriers and facilitators of utilization (drop-off and retention factors).

Throughout the development of the selfBACK DSS, patients with LBP and health care professionals have been interviewed and asked about their experience with the traditional treatment of back pain and how they usually self-manage their LBP. In addition, a panel consisting of clinicians (eg, physiotherapists, chiropractors, sports physiologists, and psychologists) provided feedback and answered a survey concerning the choice of physical exercises and the educational content. Furthermore, the selfBACK team members (eg, physiotherapists, chiropractors, exercise physiologists, and medical doctors) contributed to group discussions and the structuring of the content implemented in the selfBACK DSS. Finally, the developmental versions of the selfBACK app are continuously tested by patients and team members in iterative rounds, during which information is collected in interviews and group sessions with potential users.

## Results

The selfBACK project was launched in January 2016 and will run until the end of 2020. The version of the selfBACK DSS that will be used in the RCT will be completed in the fall of 2018. Our target population is care-seeking patients in primary care settings diagnosed with nonspecific LBP. Clinicians will identify patients who will be eligible for self-management and participation in the RCT. The recruitment of patients and data collection in the RCT will start in February 2019 with pain-related disability at 3 months as the primary outcome with additional follow-ups at 6 weeks, 6 months, and 9 months. In addition, results for the trial will be reported according to the CONSORT statement [51,52] and the extended CONSORT-EHEALTH checklist [53]. Along with publications in peer-reviewed scientific journals, the results will be disseminated to a wider audience and key stakeholders, such as patient organizations, health care professionals, and relevant policy makers, through social media and other mechanisms. Moreover, market introduction and exploitation of the results will be based on a business plan developed by the selfBACK consortium and will be ongoing throughout the project period with a strong focus toward the mobile health (mHealth) technology industry. The methodology that is being developed is expected to have wide applicability to other chronic conditions with similar conceptual elements.

## Discussion

Nonspecific LBP is a condition with large interindividual variation in symptoms, treatment responses, and outcomes and is, therefore, suitable for personalized care. Recent studies have shown that a stratified care approach for LBP results in a substantially better treatment response compared with treatment as usual [54,55] as well as the cost-effective use of health care resources [56]. The selfBACK project goes beyond the current state-of-the-art by developing a DSS that reinforces the patients’ motivation for self-management by providing a personalized plan for self-management and real-time feedback on the achievement of personal goals. Of note, the selfBACK approach is the first example of a DSS that utilizes CBR technology to tailor self-management plans for a patient with LBP.

In selfBACK, we will collect data about patients by a baseline Web-based questionnaire, an activity-detecting wearable, and weekly Q/A sessions in the selfBACK app. The obtained information will be used to personalize advice to optimize and reinforce self-management of nonspecific LBP. In addition, the collected data, along with the personalized plans for self-management, will be used to build and add new cases to the system’s knowledge repository. The system’s automatic learning component enables new knowledge and plans for self-management to be integrated into the system’s knowledge repository, whereas experiential case-based learning enables improved patient support over time. Therefore, the selfBACK system is a powerful tool to facilitate, improve, and reinforce self-management of nonspecific LBP. Furthermore, the use of the selfBACK app does not require direct medical supervision and can easily be made available to a large number of people implying a cost-effective use of resources. However, selfBACK is not intended to replace clinical care, and we will adhere to the Health On the Net Foundation (HONcode) principle [57], a code of ethics that specifies certain requirements for the quality of digital patient support. Moreover, the selfBACK system will be certified according to these principles before it is launched to users.

The educational and supportive material, general physical activity, and specific strength and flexibility exercises constitute the main components of LBP self-management. In selfBACK, the patients’ physical activity level is monitored and followed-up by data provided by an activity-detecting wearable. The wearable stores data for future synchronization and users do not need to have the phone connected at all times. The monitoring of physical activity will be accompanied by motivational textual feedback displayed on the mobile phone screen. Based on the available sensors embedded in the wearable (eg, three-axis accelerometer), different health metrics are derived, including the number of steps taken and the duration of inactivity. Moreover, we can derive the time a patient is not wearing the wearable to indicate the user pattern of the selfBACK system. Although recent studies have indicated that wearables have poor validity in estimating the energy expenditure [58,59], they have acceptable reliability [60] and validity for the step detection [61], thereby fulfilling our requirement, that is, repeated daily measurements of steps in the same individual.

Many patients find it challenging to self-manage their illness with little or no additional support and the adherence to self-management programs is commonly poor [14,15]. With the selfBACK app, we envisage that the problems of feedback, reinforcement, and the adherence to self-management can be solved by offering an evidence-based system that allows personalized follow-up and advice to patients, thereby enhancing the motivation and perception of usefulness. This speculation is supported by findings from persuasive technology research showing that the adherence to home-based exercise and self-management increases when patients receive personalized feedback, perceive the advice as evidence-based, receive reminders to stay active and exercise, and know that their adherence is being monitored [62,63]. Furthermore, empowering patients toward “self-regulatory” behavior by personal goal setting and self-monitoring increases the adherence and effectiveness of an intervention [64]. Therefore, we envisage that selfBACK will be more effective and motivating compared with the current treatment model, wherein patients are generally unsupported when self-managing nonspecific LBP.

A strength of the selfBACK approach is the strong theoretical underpinning along with the use of data-driven computer modeling to tailor advice and follow-up on self-management. Furthermore, because the advice is grounded in the system’s growing experience on the effect of plans for self-management and the accompanying symptom progression, the prediction quality of selfBACK will increase over time. Thus, the selfBACK app could potentially become a potent tool for supporting self-management in patients with LBP. Nevertheless, the risk of poor engagement or the lack of sustained participation exists. Importantly, a process evaluation will be conducted along with the RCT, enabling us to document the implementation of selfBACK, including patient experiences of using the app. This will provide clues about how patient-centered DSSs, such as selfBACK, should be designed to maximize uptake and utilization.

By adapting and advancing the state-of-the-art technology in data capture, data analysis, and proactive decision support, we will, in the selfBACK project, develop and document a DSS to be used by patients themselves to support self-management of nonspecific LBP. In line with current evidence-based recommendations and guidelines, selfBACK incorporates physical activity, education, and specific strength and flexibility exercises to improve, facilitate, and reinforce the self-management process. Furthermore, the effectiveness of selfBACK will be evaluated in a multinational RCT, targeting care-seeking patients in a primary care setting.

## Abbreviations

CBR: case-based reasoning
CONSORT: consolidated standards of reporting trials
CONSORT-EHEALTH: consolidated standards of reporting trials statement for randomized controlled trials of electronic and mobile health applications and online telehealth
COPD: chronic obstructive pulmonary disease
DSS: decision support system
EQ-5D: European Quality of Life-5 Dimensions
HONcode: Health On the Net Foundation
LBP: low back pain
RCT: randomized controlled trial
Q/A: question/answer

## Appendix

Multimedia Appendix 1 Evaluation Summary Report from the European Commission.
