# Metadata Correction: A Decision Support System to Enhance Self-Management of Low Back Pain: Protocol for the selfBACK Project

**DOI:** 10.2196/12180

**Published:** 2019-01-03

**Authors:** Paul Jarle Mork, Kerstin Bach, SelfBACK Consortium

**Affiliations:** 1 Department of Public Health and Nursing Norwegian University of Science and Technology Trondheim Norway; 2 Department of Computer Science Norwegian University of Science and Technology Trondheim Norway

The authors of the paper “A Decision Support System to Enhance Self-Management of Low Back Pain: Protocol for the selfBACK Project” (JMIR Res Protoc 2018;7(7):e167) wish to amend the authors listed on the paper. 

In the “Authors” section, the collaborators were not included and only the two main authors were listed in the authorship information. The selfBACK Consortium has been added as a group author, and the names of the contributors have now been listed in the Acknowledgments section. The full list of collaborations associated with the selfBACK Consortium can be found in Multimedia Appendix 1 of this correction notice.

The correction will appear in the online version of the paper on the JMIR website on January 3, 2019, together with the publication of this correction notice. Because this was made after submission to PubMed, PubMed Central, and other full-text repositories, the corrected article also has been resubmitted to those repositories.

## Appendix

Multimedia Appendix 1 Collaborators and their affiliations.

