# Supplementary material for: A Decision Support System to Enhance Self-Management of Low Back Pain: Protocol for the selfBACK Project
Source: JMIR Res Protoc. 2018 Jul 20;7(7):e167. doi: 10.2196/resprot.9379 (PMC6076372; doi:10.2196/resprot.9379)
Supplement: Multimedia Appendix 1 [file resprot_v7i7e167_app1.pdf]

Ref. Ares(2015)3397964 - 14/08/2015

Proposal Evaluation Form

EUROPEAN COMMISSION

Horizon 2020 - Research and Innovation Framework Programme

Evaluation Summary Report - Research and innovation actions/Innovation actions

**Call:**	H2020-PHC-2015-single-stage
**Funding scheme:**	Research and Innovation action
**Proposal number:**	689043
**Proposal acronym:**	selfBACK
**Duration (months):**	60
**Proposal title:**	A decision support system for self-management of low back pain
**Activity:**	PHC-28-2015 Predictive modelling RIA

N.	Proposer name	Country	Total Cost	%	Grant Requested	%
1	NORGES TEKNISK-NATURVITENSKAPELIGE UNIVERSITET NTNU	NO	1,941,186	39.45%	1,941,186	39.45%
2	UNIVERSITY OF GLASGOW	UK	412,566	8.38%	412,566	8.38%
3	THE ROBERT GORDON UNIVERSITY	UK	565,446	11.49%	565,446	11.49%
4	Kiolis	FR	793,093	16.12%	793,093	16.12%
5	DET NATIONALE FORSKNINGSCENTER FORARBEJDSMILJO	DK	297,360	6.04%	297,360	6.04%
6	Health Leads BV	NL	346,875	7.05%	346,875	7.05%
7	SYDDANSK UNIVERSITET	DK	564,345	11.47%	564,345	11.47%
	Total:		4,920,871		4,920,871	

Abstract:
The recent global burden of disease study showed that low back pain (LBP) is the most significant contributor to disability in Europe. Most patients seen in primary care with LBP have non-specific LBP (≥85%), i.e., pain that cannot reliably be attributed to a specific disease/pathology. LBP is the fourth most common diagnosis seen in primary care (after upper respiratory infection, hypertension, and coughing). Self-management in the form of physical activity and strength/stretching exercises constitutes the core component in the management of non-specific LBP; however, adherence to self-management challenging due to lack of feedback and reinforcement. This project aims to develop a decision support system - SELFBACK - that will be used by the patient him/herself to facilitate, improve and reinforce self-management of LBP. Specifically, SELFBACK will be designed to assist the patient in deciding and reinforcing the appropriate actions to manage own LBP after consulting a health care professional in primary care. The decision support will be conveyed to the patient via a smartphone app in the form of advice for self-management. The advice will be tailored to each patient based on the symptom state, symptom progression, the patients goal-setting, and a range of patient characteristics including information from a physical activity-detecting wristband worn by the patient. The second part of the project will evaluate the effectiveness of SELFBACK in a randomized controlled trial using pain-related disability as primary outcome. We envisage that patients who use SELFBACK will have 20% reduction in pain-related disability at 9 months follow-up compared to patients receiving treatment as usual. Process evaluation will be carried out as an integrated part of the trial to document the implementation and map the patients' satisfaction with SELFBACK. A business plan with a targeted commercialisation strategy will be developed to transfer the SELFBACK technology into the market.

Evaluation Summary Report

Evaluation Result

Total score: 14.50 (Threshold: 12)

Form information

SCORING

Scores must be in the range 0-5.

Interpretation of the score:

*0 The **proposal fails to address the criterion** or cannot be assessed due to missing or incomplete information.*

***1 Poor.** The criterion is inadequately addressed, or there are serious inherent weaknesses.*

***2 Fair.** The proposal broadly addresses the criterion, but there are significant weaknesses.*

***3 Good.** The proposal addresses the criterion well, but a number of shortcomings are present.*

***4 Very good.** The proposal addresses the criterion very well, but a small number of shortcomings are present.*

***5 Excellent.** The proposal successfully addresses all relevant aspects of the criterion.Any shortcomings are minor.*

Criterion 1 - Excellence

Score: 5.00 (Threshold: 4/5.00 , Weight: 100.00%)

Note: The following aspects will be taken into account, to the extent that the proposed work corresponds to the topic description

in the work programme. If a proposal is partly out of scope, this must be reflected in the scoring, and explained in the comments.
Clarity and pertinence of the objectives
Credibility of the proposed approach
Soundness of the concept, including trans-disciplinary considerations, where relevant
Extent that proposed work is ambitious, has innovation potential, and is beyond the state of the art (e.g. ground-breaking objectives, novel concepts and approaches)

The proposal is excellent and relevant to the call. The objectives to build a predictive system based on computer modelling, providing decision support for patients with low back pain are clearly defined.

The proposal is highly credible and justified by a well-documented approach including investigation, evolution, treatment, and prevention.

The concept is sound and clearly describes the previously developed ontology to build the DSS. Trans-disciplinary considerations are well covered, including both the medical and ICT domains.

The proposed research is novel and very ambitious and goes beyond the state of the art, comprising several innovative features which are well documented.

The case-based reasoning cycle approach previously developed will be used as a model for the DSS and can be translated to several similar medical problems. In addition, the hardware requirements (wristband) to work alongside the DSS are simple, which will facilitate stakeholder adoption.

Criterion 2 - Impact

Score: **4.50** (Threshold: 4/5.00 , Weight: 100.00%)

Note: The following aspects will be taken into account, to the extent to which the outputs of the project should contribute at the European and/or International level:
The expected impacts listed in the work programme under the relevant topic
Enhancing innovation capacity and integration of new knowledge
Strengthening the competitiveness and growth of companies by developing innovations meeting the needs of European and global markets, and where relevant, by delivering such innovations to the markets
Any other environmental and socially important impacts (not already covered above)
Effectiveness of the proposed measures to exploit and disseminate the project results (including management of IPR), to communicate the project, and to manage research data where relevant

The expected impacts listed in the work programme are all addressed, with the aim to improve patient self-management of low back pain and increase prevention by using predictive modelling.

The integration of new knowledge and improving innovation capacity is assured by the greater insight and information on patient use of portable devices to monitor various health parameters. Further, the open source approach will significantly add to the adaptation of the developed tools.

The proposal will develop innovation capacity and will contribute to the increased competitiveness of partner companies. Given the prevalence of this illness, a high social impact is expected for EU citizens by reducing the consumption of analgesics and a corresponding increase in the quality of life of patients with low back pain.

The dissemination plan is ambitious, comprising not only classical approaches, but also the involvement of patient organizations, insurance companies, national and local governments. The proposal presents a general exploitation strategy, as well as a systematized presentation of the business plan.

IPR management and protection are appropriately addressed, as is the research management plan. However, there are no comments about the security of the data stored on the individual devices.

Criterion 3 - Quality and efficiency of the implementation

Score: **5.00** (Threshold: 3/5.00 , Weight: 100.00%)

Note: The following aspects will be taken into account:
Coherence and effectiveness of the work plan, including appropriateness of the allocation of tasks and resources
Complementarity of the participants within the consortium (when relevant)
Appropriateness of the management structures and procedures, including risk and innovation management

The work-plan is coherent and clearly organized. The work-packages are described professionally and include all relevant details about the objectives, tasks, deliverables and contribution of partners. The milestones are well-defined, for example the basic system will be ready half-way through the project, allowing for early initiation of randomised trials.

Resources are well allocated and commensurate with the work described.

The consortium is complementary and provides a good balance of academic and industry partners covering all areas of necessary expertise to achieve the objectives. The inclusion of an industry partner at the end of the technology chain increases the likelihood for the DSS to be implemented.

The management structure is appropriate for this size of consortium. All responsibilities are well defined, with clear mechanisms for decision, conflict resolution and communication. Risk management is also well addressed, including mitigation measures.

Operational Capacity

Status: **Operational Capacity: Yes**

Not provided

Proposal content corresponds, wholly or in part, to the topic description against which it is submitted, in the relevant work programme part

Status: **Yes**

Not provided

Overall comments

Not provided
