# Supplementary material for: Metadata Correction: A Decision Support System to Enhance Self-Management of Low Back Pain: Protocol for the selfBACK Project
Source: JMIR Res Protoc. 2019 Jan 3;8(1):e12180. doi: 10.2196/12180 (PMC6682264; doi:10.2196/12180)
Supplement: Multimedia Appendix 1 [file resprot_v8i1e12180_app1.pdf]

Partner	Academic title	Full name	Affiliation	Affiliation (if more than one)
NFA	MSc, PhD	Charlotte Diana Nørregaard Rasmussen	National Research Centre for the Working Environment, Copenhagen, Denmark	
NFA	MSc	Malene Jagd Svendsen	National Research Centre for the Working Environment, Copenhagen, Denmark	
NFA	MSc, PhD	Andreas Holtermann	National Research Centre for the Working Environment, Copenhagen, Denmark	Department of Sports Science and Clinical Biomechanics, University of Southern Denmark, Odense, Denmark
GLA	BSc (Hons), PGDip, PhD	Barbara I Nicholl	Institute of Health & Wellbeing, University of Glasgow, Scotland	
GLA	MBChB, MD, DRCOG, FRCGP	Frances S Mair	Institute of Health & Wellbeing, University of Glasgow, Scotland	
GLA	MA (Hons)	Karen Wood	Institute of Health & Wellbeing, University of Glasgow, Scotland	
UoSD	MSc, PhD	Mette Jensen Stochkendahl	Department of Sports Science and Clinical Biomechanics, University of Southern Denmark, Odense, Denmark	Nordic Institute of Chiropractic and Clinical Biomechanics, Odense, Denmark
UoSD	MSc, PhD	Karen Søgaard	Department of Sports Science and Clinical Biomechanics, University of Southern Denmark, Odense, Denmark	Occupational and Environmental Medicine, University Hospital, Odense, Denmark
UoSD	DC, PhD	Jan Hartvigsen	Department of Sports Science and Clinical Biomechanics, University of Southern Denmark, Odense, Denmark	Nordic Institute of Chiropractic and Clinical Biomechanics, Odense, Denmark
UoSD	PT, MSc, PhD	Per Kjær	Department of Sports Science and Clinical Biomechanics, University of Southern Denmark, Odense, Denmark	
UoSD	MSc, PhD	Louise Fleng Sandal	Department of Sports Science and Clinical Biomechanics, University of Southern Denmark, Odense, Denmark	
UoSD	MSc, PhD	Gisela Sjøgaard	Department of Sports Science and Clinical Biomechanics, University of Southern Denmark, Odense, Denmark	
HLE	PT	Peter Vermeiren	Health Leads BV, Bussum, Netherlands	
HLE	MSc	Marco Pieterse	Health Leads BV, Bussum, Netherlands	
RGU	MSc, PhD	Nirmalie Wiratunga	School of Computing Science and Digital Media, Robert Gordon University, Aberdeen, Scotland	
RGU	MSc, PhD	Sadiq Sani	School of Computing Science and Digital Media, Robert Gordon University, Aberdeen, Scotland	

RGU	PT, MSc, PhD	Kay Cooper	School of Health Sciences,<br>Robert Gordon University, Aberdeen, Scotland	
RGU	MSc, PhD	Stewart Massie	School of Computing Science and Digital Media,<br>Robert Gordon University, Aberdeen, Scotland	
TRX	Diploma in Export and Technology	Søren Kleberg	Trade eXpansion Aps, Tommerup, Denmark	
TRX	AP Degree in Marketing Management	Yvonne A. Küttemann	Trade eXpansion Aps, Tommerup, Denmark	
TRX	BSc	Rasmus Faddersbøll	Trade eXpansion Aps, Tommerup, Denmark	
TRX	BSc	Tony Dieu	Trade eXpansion Aps, Tommerup, Denmark	
TRX	PBA	Christian Lodberg Jensen	Trade eXpansion Aps, Tommerup, Denmark	
NTNU	MSc, PhD	Paul Jarle Mork	Department of Public Health and Nursing, Faculty of Medicine and Health Sciences, Norwegian University of Science and Technology (NTNU), Trondheim, Norway	
NTNU	MSc, PhD	Kerstin Bach	Department of Computer Science, Faculty of Information Technology and Electrical Engineering, Norwegian University of Science and Technology (NTNU), Trondheim, Norway	
NTNU	PT, MSc, PhD	Ottar Vasseljen	Department of Public Health and Nursing, Faculty of Medicine and Health Sciences, Norwegian University of Science and Technology (NTNU), Trondheim, Norway	
NTNU	MSc, PhD	Tom Ivar Lund Nilsen	Department of Public Health and Nursing, Faculty of Medicine and Health Sciences, Norwegian University of Science and Technology (NTNU), Trondheim, Norway	
NTNU	MSc	Tomasz Szczepanski	Department of Computer Science, Faculty of Information Technology and Electrical Engineering, Norwegian University of Science and Technology (NTNU), Trondheim, Norway	
NTNU	MSc, PhD	Agnar Aamodt	Department of Computer Science, Faculty of Information Technology and Electrical Engineering, Norwegian University of Science and Technology (NTNU), Trondheim, Norway	
NTNU	MSc, PhD	Ilya Ashikhmin	Department of Computer Science, Faculty of Information Technology and Electrical Engineering, Norwegian	

			University of Science and Technology (NTNU), Trondheim, Norway	
NTNU	MSc, PhD	Morten Villumsen	Department of Public Health and Nursing, Faculty of Medicine and Health Sciences, Norwegian University of Science and Technology (NTNU), Trondheim, Norway	
